# Diagnostic and Therapeutic Challenges in a Case of Achalasia Masquerading as Gastroesophageal Reflux Disease (GERD)

**DOI:** 10.7759/cureus.91020

**Published:** 2025-08-26

**Authors:** Rahul S Nanduri, Alekhya Gurram, Noah Karnath, Bernard Karnath

**Affiliations:** 1 Department of Internal Medicine, John Sealy School of Medicine, University of Texas Medical Branch, Galveston, USA

**Keywords:** achalasia, botox, dysphagia, gastroesophageal reflux disease (gerd), lower esophageal sphincter (les), myotomy

## Abstract

Achalasia is a rare swallowing disorder that occurs due to the deterioration of the inhibitory neurons that control the lower esophageal sphincter (LES). Achalasia shares many clinical characteristics and symptoms with gastroesophageal reflux disease (GERD), a common process involving reflux of stomach acid into the esophagus, causing difficulties in differentiating diagnoses for therapeutic management. We report the case of a 49-year-old male patient with a history of chronic GERD who presented with dysphagia. Despite proton pump inhibitor (PPI) therapy, the patient developed worsening dysphagia. Esophagogastroduodenoscopy (EGD) suggested a diagnosis of achalasia, later confirmed with barium esophagram and manometry. Due to worsening symptoms and concerns about surgical fitness, the patient underwent bridge Botox therapy. However, further deterioration necessitated surgical myotomy. This case highlights the importance of a full dysphagia evaluation, including barium esophagram and EGD. This case also highlights the treatment of achalasia, which may include Botox injections as bridge therapy and myotomy for complete resolution.

## Introduction

Achalasia is a rare, smooth muscle swallowing disorder characterized by impaired relaxation of the lower esophageal sphincter (LES) due to inhibitory neuronal damage and absent peristalsis. The peak age of incidence is 30 to 60 years, with dysphagia, painful swallowing, and regurgitation as the most common presenting symptoms [1]. The prevalence is estimated to be seven to 32 cases per 100,000 individuals [2]. Most cases are idiopathic, while some are secondary to Chagas disease, esophageal infiltration by gastric carcinoma, eosinophilic gastroenteritis, lymphoma, viral infections, or neurodegenerative disorders [1]. Its rarity, overlapping symptoms with gastroesophageal reflux disease (GERD), and varied treatment response pose diagnostic and therapeutic challenges. The reported case presented atypically, with potentially coexisting GERD and achalasia in the setting of pre-existing chronic reflux, for which Botox bridge therapy was used prior to myotomy.

## Case presentation

The patient was a 49-year-old man with a history of morbid obesity, cardiomyopathy, and GERD presenting with difficulty swallowing that was worse with solid foods and a sensation of food stuck in his throat. The patient reported compliance with 30 mg lansoprazole for 10 years for his GERD, but stated that he was taking medication only 10 minutes before meals, instead of 30 minutes before as recommended. A flare of reflux symptoms was suspected, and the patient was educated to take his medication appropriately, 30 minutes before meals.

**Figure 1:**
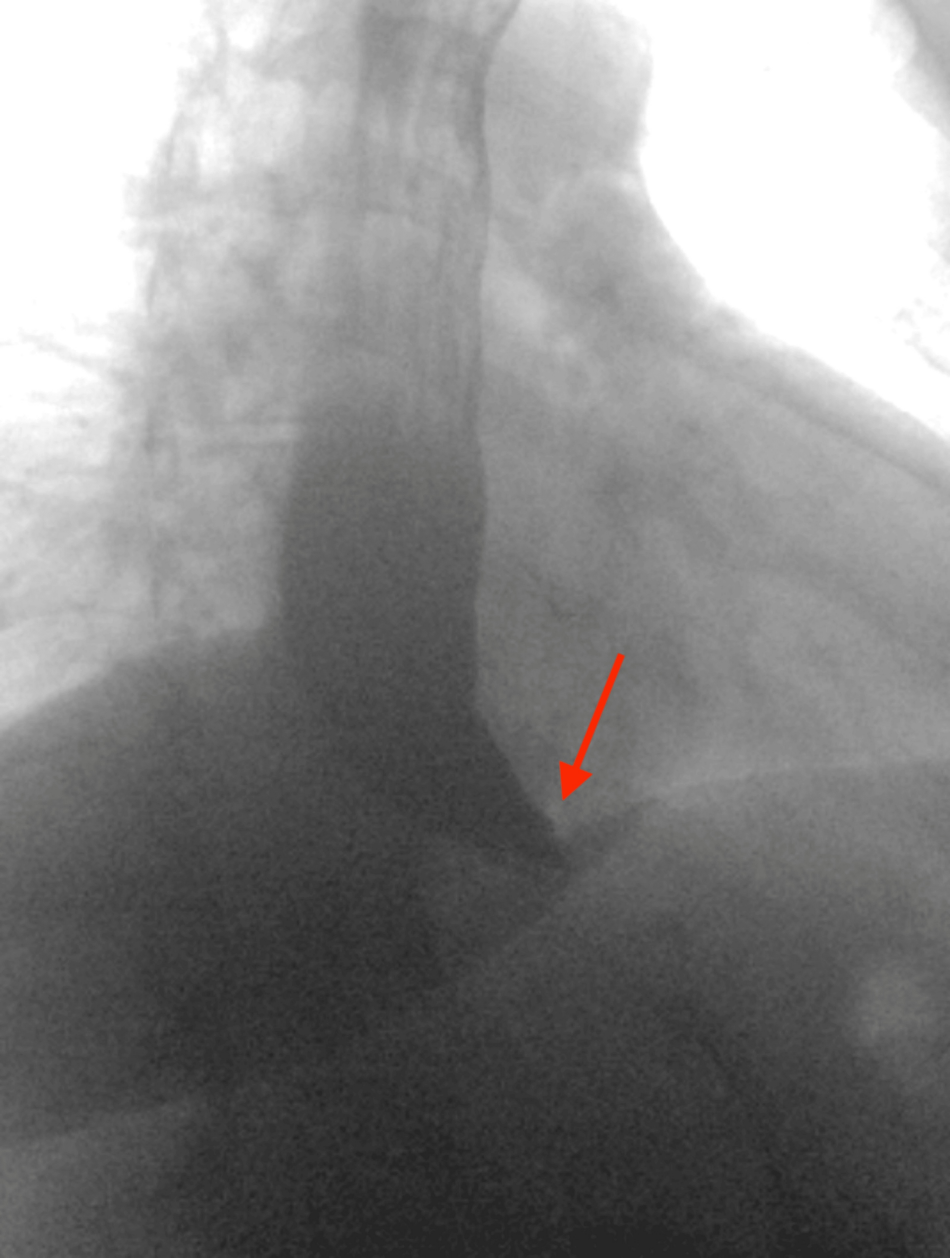
A barium swallow test showing dilation of the proximal esophagus and increased lower esophageal sphincter (LES) tone, with a classic “bird beak” appearance. Slow emptying and intermittent retention of barium at the LES is also noted.

A barium swallow test was scheduled one week later, which revealed severe LES spasm, intermittent retention of barium in the esophagus, and slow emptying. The patient reported that lansoprazole was not alleviating his reflux symptoms, so his medication regimen was changed to 40 mg omeprazole daily, with the addition of Maalox 30 mL, Donnatal 15 mL, and 2% lidocaine cocktail. Although a referral to gastroenterology (GI) was placed, the patient chose to pursue GI evaluation at an outside institution due to cost barriers. Two months after his first barium esophagram, the patient underwent another barium swallow study (Figure 1), manometry, and esophagogastroduodenoscopy (EGD) at the outside institution, which confirmed achalasia. The patient was recommended for surgical myotomy pending clearance for surgical intervention due to pre-existing cardiomyopathy and obesity. The patient was also advised to reduce his BMI from 47 to 35 kg/m² to optimize surgical outcomes.

**Figure 2:**
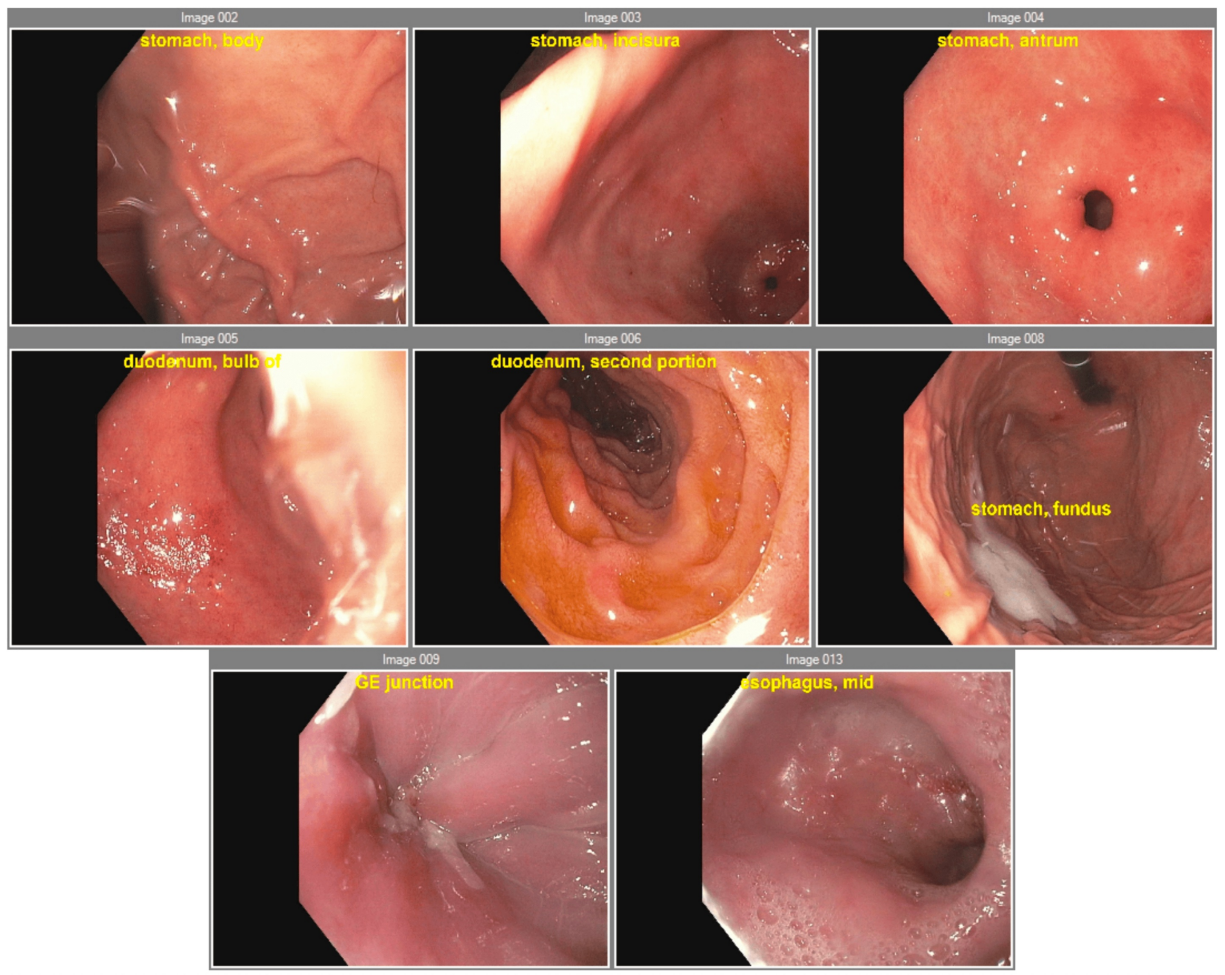
Endoscopic pictures taken during the esophagogastroduodenoscopy (EGD) showing a mildly tortuous esophagus, dilation in the mid and distal esophagus, stenosis at the gastroesophageal junction, and mild gastritis in the antrum of the stomach

Two weeks later, the patient presented with acutely exacerbated symptoms with minimal tolerance of liquids. Due to failure to meet pre-operative goals, the patient underwent EGD with Botox injection to the LES as bridge therapy (Figure 2).

However, persistent deterioration necessitated surgical myotomy despite suboptimal surgical fitness. The patient underwent an uncomplicated laparoscopic Heller myotomy with anterior fundoplication. One month later, the patient endorsed significant recovery from his dysphagia, although he noted persistent reflux symptoms managed through his prescribed medication regimen.

## Discussion

Due to overlapping symptoms, achalasia and GERD are difficult to differentiate, especially in this case due to the concurrent presence of both pathologies [1, 2]. The cardinal symptoms of achalasia are dysphagia, regurgitation, chest pain, and weight loss [2, 3]. Classic symptoms of GERD include regurgitation and heartburn, but GERD may also present with atypical symptoms or extra-esophageal manifestations [4]. Almost 48% of GERD patients present with dysphagia, so this cannot necessarily be used to favor achalasia as a diagnosis over GERD, although achalasia more commonly presents with progressive dysphagia and GERD with intermittent dysphagia [5]. Similarly, although heartburn is not a cardinal symptom of achalasia, many patients with achalasia report heartburn [6]. Thorough history taking is necessary to favor one diagnosis over the other. Although regurgitation may be present in both GERD and achalasia, regurgitation of undigested food can suggest achalasia, while acidic, bitter regurgitation not containing food may suggest GERD [4-6]. GERD symptoms may occur soon after meals or when supine, while achalasia symptoms tend to occur hours after eating [2, 4].

Suspicion of acute GERD exacerbation in this patient was increased because he was taking his medication only 10 minutes prior to his meals. Proton pump inhibitor (PPI) medication should be taken 30-60 minutes before meals for adequate dosing prior to proton pump activation [7]. However, even when medication is being taken suboptimally, clinicians must consider achalasia in patients with dysphagia and GERD symptoms. This case was further complicated by social determinants of health that delayed a full work-up. Although the patient underwent a barium esophagram that suggested achalasia, he was lost to follow-up from our institution. As a result, no therapeutic management was pursued for several months, which potentially predisposed symptomatic progression. Delaying testing or management can allow achalasia to progress from solid to mixed (solid and liquid) dysphagia, so early intervention must be pursued.

Achalasia treatment may include Heller's myotomy, Botox, and endoscopic balloon dilation [8]. Heller’s myotomy is considered first-line in cases where a surgical anti-reflux procedure may also be indicated due to high effectiveness, high remission rate, and low relapse rate [9]. Per-oral endoscopic myotomy (POEM) is a less invasive alternative to Heller's myotomy that can also be considered first-line management; however, studies show that the incidence of postoperative reflux is higher after POEM as compared to Heller's myotomy with fundoplication, based on reflux symptoms, endoscopic monitoring, and pH monitoring [10]. Botox injection often requires further intervention after six to seven months, which limits long-term effectiveness [9]. Balloon dilation is more effective than Botox injection but less effective than Heller’s myotomy [11,12]. In the presented patient, the goal of treatment was Heller’s myotomy due to disease severity and the patient’s comorbidity profile. However, the sudden worsening of symptoms with failure to meet preoperative goals necessitated Botox injection as a temporizing measure for immediate symptomatic relief.

In this case, achalasia may have developed due to chronic GERD. Although a causal relationship has yet to be strongly defined, several reports hypothesize that chronic GERD could be a risk factor for achalasia development [13, 14]. LES muscle specimens from reflux patients show morphological and metabolic changes, such as inflammatory infiltration of the myenteric plexus and structural protein hypertrophy, that indicate achalasia-like changes [15, 16]. The development of achalasia may be a physiological defense mechanism against chronic acid exposure in patients with longstanding reflux disease [17]. Suspicion for achalasia should be higher in patients with chronic GERD upon complaints of dysphagia. Although the patient experienced persistent reflux symptoms in this case, laparoscopic Heller’s myotomy with 360-degree fundoplication as surgical intervention may minimize the risk of postoperative reflux disease [18].

## Conclusions

Achalasia is an important consideration in a patient with dysphagia and reflux. This case illustrates the complex technicalities of differentiating between achalasia and GERD. A barium esophagram must be scheduled at the earliest to prevent disease progression due to a delay in intervention. In addition, when treating achalasia, decision-making when considering Botox injections and myotomy must incorporate a holistic view of the patient’s condition to optimize outcomes. This case also highlights the necessity of ongoing clinical monitoring of patients with chronic diseases like GERD to monitor for medication noncompliance as well as potential overlapping symptoms that could suggest another superimposed disease process.
